# Cancer incidence and cause-specific mortality in patients with metal-on-metal hip replacements in Finland: A population-based study with a mean follow-up of 4.6 (1–11) years

**DOI:** 10.3109/17453674.2013.878830

**Published:** 2014-02-25

**Authors:** Keijo T Mäkelä, Tuomo Visuri, Pekka Pulkkinen, Antti Eskelinen, Ville Remes, Petri Virolainen, Mika Junnila, Eero Pukkala

**Affiliations:** ^1^Department of Orthopaedics and Traumatology,Surgical Hospital, Turku University Hospital,Turku; ^2^Hjelt Institute, Helsinki University,Helsinki; ^3^Coxa Hospital for Joint Replacement, Tampere; ^4^Department of Orthopaedics and Traumatology,Peijas Hospital, Helsinki University Central Hospital,Helsinki; ^5^School of Health Sciences,University of Tampere, Tampere and the Finnish Cancer Registry, Institute for Statistical and Epidemiological Cancer Research, Helsinki,Finland.

## Abstract

**Background and purpose:**

Metal-on-metal hip implants have been widely used, especially in the USA, Australia, England and Wales, and Finland. We assessed risk of death and updated data on the risk of cancer related to metal-on-metal hip replacements.

**Patients and methods:**

A cohort of 10,728 metal-on-metal hip replacement patients and a reference cohort of 18,235 conventional total hip replacement patients were extracted from the Finnish Arthroplasty Register for the years 2001–2010. Data on incident cancer cases and causes of death until 2011 were obtained from the Finnish Cancer Registry and Statistics Finland. The relative risk of cancer and death were expressed as standardized incidence ratio (SIR) and standardized mortality ratio (SMR). SIR/SIR ratios and SMR/SMR ratios, and Poisson regression were used to compare the cancer risk and the risk of death between cohorts.

**Results:**

The overall risk of cancer in the metal-on-metal cohort was not higher than that in the non-metal-on-metal cohort (RR = 0.91, 95% CI: 0.82–1.02). The risk of soft-tissue sarcoma and basalioma in the metal-on-metal cohort was higher than in the non-metal-on-metal cohort (SIR/SIR ratio = 2.6, CI: 1.02–6.4 for soft-tissue sarcoma; SIR/SIR ratio = 1.3, CI: 1.1–1.5 for basalioma). The overall risk of death in the metal-on-metal cohort was less than that in the non-metal-on-metal cohort (RR = 0.78, CI: 0.69–0.88).

**Interpretation:**

The overall risk of cancer or risk of death because of cancer is not increased after metal-on-metal hip replacement. The well-patient effect and selection bias contribute substantially to the findings concerning mortality. Arthrocobaltism does not increase mortality in patients with metal-on-metal hip implants in the short term. However, metal-on-metal hip implants should not be considered safe until data with longer follow-up time are available.

Metal-on-metal hip implants have been widely used, especially in the USA, Australia, England and Wales, and Finland (AOANJRR 2010, NJR 2011, Cohen 2012, Seppänen et al. 2012). The theoretical health risks related to chronically elevated blood metal ion concentrations induced by abnormal wear and corrosion of the metal-on-metal implants—apart from local symptoms around the failing implant—include systemic symptoms of poisoning (Steens et al. 2006, Oldenburg et al. 2009, Rizzetti et al. 2009, Tower 2010, 2012, Mao et al. 2011, Sotos and Tower 2013, Zyviel et al. 2013) and carcinogenesis (Mäkelä et al. 2012, Smith et al. 2012, Brewster et al. 2013). Systemic metal ion toxicity cases due to a failed hip replacement are rare. However, there have been several recent reports of systemic cobalt toxicity following revision of fractured ceramic components, and also in patients with a failed metal-on-metal hip replacement (Steens et al. 2006, Oldenburg et al. 2009, Rizzetti et al. 2009, Tower 2010, 2012, Mao et al. 2011, Sotos and Tower 2013, Zyviel et al. 2013). Possible clinical findings include fatigue, weakness, hypothyroidism, cardiomyopathy, polycythemia, visual and hearing impairment, cognitive dysfunction, and neuropathy. Fatal cardiomyopathy due to systemic cobalt toxicity after hip replacement has been reported (Zyviel et al. 2013).

Metal debris from hip replacement may be associated with chromosomal aberrations and DNA damage (Case et al. 1996, Bonassi et al. 2000, Daley et al. 2004). However, the risk of cancer is not increased after conventional metal-on-polyethylene total hip replacement or after first-generation metal-on-metal total hip arthroplasty (Visuri et al. 1996, 2010a). The short-term overall cancer risk after modern metal-on-metal hip arthroplasty is not increased either (Mäkelä et al. 2012, Smith et al. 2012, Brewster et al. 2013). However, recent linkage studies of overall cancer risk are based on hospital episode statistics, which may have less quality assurance than cancer registry data (Smith et al. 2012, Brewster et al. 2013). Annual updating of cancer registry data concerning the metal-on-metal issue is advisable.

In this paper, we update our earlier published results on risk of cancer (Mäkelä et al. 2012) and give an assessment of the overall and cause-specific mortality in primary metal-on-metal and non-metal-on-metal hip replacement patients who were operated on from 2001 to 2010, by combining data from the Finnish Arthroplasty Register, the Population Register Centre, and the Finnish Cancer Registry. The reason for this early updating of the cancer data was to be able to detect a cancerogenic effect of metal-on-metal implants as early as possible.

## Patients and methods

The metal-on-metal cohort consisted of 10,728 patients and the non-metal-on-metal cohort consisted of 18,235 patients (Mäkelä et al. 2012). For details, see Mäkelä et al. (2012). None of the subjects were lost to follow-up.

### Follow-up and statistical analysis

The patients were followed up from the date of the first hip replacement until death, or until December 31, 2011. The data from the Finnish Arthroplasty Register were linked with the data from the Finnish Cancer Registry (Teppo et al. 1994) using the unique personal identity codes of the patients. Dates of death or emigration and causes of death were obtained from Statistics Finland. The Finnish Cancer Registry covers more than 99% of all cancer cases in Finland (Teppo et al. 1994). Determination of the cause of death is based on the medical or forensic evidence, which provides the grounds for issue of a death certificate. Forensic determination of the cause of death may be necessary if the death is not the result of an illness, if it is accidental or violent, or if it is caused by a treatment procedure or an occupational disease. In most other cases, the death certificate is based on medical evidence (Statistics Finland 1999).

The numbers of observed cases for each cancer category and for each cause of death category and person-years at follow-up were stratified by sex, calendar period (2001–2005 and 2006–2011), 5-year age group, and follow-up time since the operation (< 5 years and ≥ 5 years). The expected number of cancer and the number of patients expected to die from each cause were calculated by applying the number of person-years in each stratum to the corresponding cancer incidence rate and mortality rate, respectively, in the Finnish population. The relative risk of cancer or death was expressed as the ratio of the observed and expected number of cases, i.e. standardized incidence ratio (SIR) or standardized mortality ratio (SMR). Risk ratio of the 2 SIRs (SIR/SIR ratio) was used for comparison of the metal-on-metal cohort and the non-metal-on-metal cohort. The 95% confidence intervals (CIs) were defined assuming that the number of observed cases followed a Poisson distribution. A Poisson regression analysis to further compare the cancer risk in the metal-on-metal and non-metal-on-metal cohorts was performed for all cancers and for colon cancer, prostate cancer, lung cancer, and basalioma. These cancer types were included in the regression analysis because the number of cases was sufficient. A Poisson regression model was also used for comparison of the risk of death in the metal-on-metal cohort and in the non-metal-on-metal cohort. In Poisson regression analyses, age was stratified in 30-year categories and follow-up time was stratified in 3 categories (for cancer: < 2, 2–5, and > 5 years since the operation; and for death: < 1, 1–5, and > 5 years since the operation). In addition, sex was added in the model.

## Results

The metal-on-metal cohort gave 48,978 person-years and the non-metal-on-metal cohort gave 108,904 person-years (Table 1). The mean follow-up of the metal-on-metal cohort was 4.6 (1–11) years and that of the non-metal-on-metal cohort was 6.0 (1–11) years.

**Table 1.** Number of patients (n) according to age at operation, and number of person-years according to the age at follow-up. The non-metal-on-metal cohort consisted of implants with metal-on-polyethylene, ceramic-on-polyethylene, and ceramic-on-ceramic bearing surfaces

	Metal-on-metal (MoM) cohort				Non-metal-on-metal cohort			
	Men		Women		Men		Women	
Age	n	Person-years	n	Person-years	n	Person-years	n	Person-years
< 20	6	14	3	16	–	–	–	–
20–29	25	101	16	56	4	17	7	23
30–39	158	435	67	236	30	101	20	87
40–49	741	2,833	468	1,569	143	616	157	491
50–59	2,275	8,516	1,642	6,226	850	3,327	922	3,711
60–69	2,257	11,955	1,581	8,383	2,260	11,140	2,739	12,557
70–79	762	4,599	594	3,263	3,044	19,193	5,394	29,603
≥ 80	65	430	48	346	697	7,844	1,953	20,193
								
Total	6,289	28,884	4,419	20,094	7,028	42,239	11,192	66,665

### Cancer incidence

The overall cancer risk in the metal-on-metal cohort was not higher than that in the Finnish population (Table 2). In the regression model, the overall cancer risk in the metal-on-metal cohort was not any higher than that in the non-metal-on-metal cohort (RR = 0.9, CI: 0.8–1.0; p = 0.1).

**Table 2.** Observed numbers of cancer cases, the expected numbers of cancer cases approximated from the Finnish population, and standardized incidence ratios with 95% confidence intervals—according to site—are given for the metal-on-metal cohort and for the non-metal-on-metal cohort. The latter cohort consisted of implants with metal-on-polyethylene, ceramic-on-polyethylene, and ceramic-on-ceramic bearing surfaces

	MoM cohort				Non-MoM cohort			
Primary site	Obs	Exp	SMR	95% CI	Obs	Exp	SMR	95% CI
All sites	497	534	0.93	0.85–1.01	1952	1908	1.02	0.98–1.06
Stomach	13	12	1.08	0.57–1.84	57	53	1.07	0.81–1.39
Colon	20	29	0.69	0.42–1.05	118	132	0.89	0.74–1.06
Lung	32	53	0.61	0.41–0.85 **^b^**	126	181	0.70	0.58–0.82 **^c^**
Corpus uteri	14	13	1.08	0.59–1.81	61	58	1.05	0.80–1.35
Prostate	135	124	1.09	0.91–1.27	334	334	1.00	0.90–1.10
Kidney	16	18	0.91	0.52–1.47	62	61	1.01	0.77–1.29
Bladder	11	18	0.61	0.30–1.08	80	71	1.12	0.89–1.39
Soft-tissue sarcoma	7	3	2.14	0.86–4.40	11	12	0.95	0.47–1.69
Non-Hodgkin lymphoma	17	21	0.82	0.48–1.31	81	73	1.11	0.88–1.38
Hodgkin lymphoma	1	1	0.79	0.02–4.40	1	3	0.35	0.01–1.92
Multiple myeloma	5	6	0.79	0.26–1.83	28	28	1.02	0.68–1.47
Leukemia	7	9	0.75	0.30–1.55	38	38	1.00	0.71–1.37
Melanoma	21	19	1.09	0.67–1.65	73	56	1.30	1.02–1.63 **^a^**
Basalioma	178	132	1.35	1.16–1.55 **^c^**	626	586	1.07	0.99–1.15

Obs: observed number of cancer cases; Exp: expected number of cancer cases from the Finnish population; SIR: standardized incidence ratio; CI: confidence interval.

**^a^** p < 0.05,

**^b^** p < 0.01,

**^c^** p < 0.001.

Risk of basalioma in the metal-on-metal cohort was higher than in the Finnish population (SIR = 1.4, CI: 1.2–1.6; p < 0.001) (Table 2). Risk of basalioma in the metal-on-metal cohort was also higher than in the non-metal-on-metal cohort, both in the non-stratified analysis (SIR/SIR ratio = 1.3, CI: 1.1–1.5) (Table 3) and in the stratified regression analysis (RR = 1.3, CI: 1.1–1.5; p = 0.01).

**Table 3.** SIR/SIR ratios for the metal-on-metal group and the non-metal-on-metal group (consisting of implants with metal-on-polyethylene, ceramic-on-polyethylene, and ceramic-on-ceramic bearing surfaces) with 95% confidence intervals, according to site

Primary site	SIR/SIR ratio	95% CI
All sites	0.91	0.82–1.00
Stomach	1.01	0.56–1.82
Colon	0.77	0.48–1.23
Lung	0.87	0.59–1.28
Prostate	1.09	0.89–1.33
Kidney	0.87	0.51–1.51
Bladder	0.54	0.29–1.01
Uterus	1.02	0.58–1.82
Soft tissue	2.55	1.02–6.36
Non-Hodgkin lymphoma	0.73	0.44–1.22
Hodgkin lymphoma	3.00	0.31–28.7
Multiple myeloma	0.83	0.33–2.09
Leukemia	0.78	0.35–1.71
Melanoma	0.85	0.52–1.37
Skin, basal cell carcinoma	1.26	1.07–1.49

SIR: Standardized incidence ratio.

The SIR of skin melanoma in the metal-on-metal cohort was 1.1 (CI: 0.67–1.7) and that in the non-metal-on-metal cohort was 1.3 (CI: 1.0–1.7) relative to the Finnish population (Table 2). Risk of melanoma in the metal-on-metal cohort was not any higher than in the non-metal-on-metal cohort, both in the non-stratified regression analysis (Table 3) and in the stratified regression analysis (RR = 0.8, CI: 0.5–1.4; p = 0.4).

7 soft-tissue sarcomas were found in the metal-on-metal cohort during the follow-up period (SIR = 2.1, CI: 0.9–4.4) (Table 2). The risk of soft-tissue sarcoma in the metal-on-metal cohort was higher than in the non-metal-on-metal cohort (RR = 2.6, CI: 1.0–6.4) (Table 3). 2 new soft-tissue sarcomas were diagnosed in 2011 in the metal-on-metal cohort, after the closing year (2010) of the earlier analysis of the same cohort (Mäkelä et al. 2012). A 66-year-old male patient with a Biomet ReCap-Magnum THA inserted in both hips in 2005 was operated for a retroperitoneal low-grade liposarcoma fixed to the right ileopsoas muscle (8 kg in weight and 30 cm in diameter). A 64-year-old male patient with an ASR resurfacing inserted in his left hip in 2004 was operated for a low-grade liposarcoma of the left adductor lodge in 2011.

In the regression analysis, the risks of lung cancer (RR = 0.9, CI: 0.7–1.3; p = 0.7), prostate carcinoma (RR = 1.1, CI: 0.9–1.4; p = 0.4), and colon carcinoma (RR = 1.0, CI: 0.6–1.7; p = 1.0) were not significantly different in the metal-on-metal cohort and the non-metal-on-metal cohort.

### Mortality

The all-cause SMR was 0.65 (CI: 0.58–0.71) for the metal-on-metal cohort and 0.72 (CI: 0.70–0.75) for the non-metal-on-metal cohort, as compared to the Finnish population (Table 4). The overall risk of death in the metal-on-metal cohort was less than that in the non-metal-on-metal cohort (RR = 0.78, CI: 0.69–0.88; p < 0.001). SMRs for deaths are presented in the Figure. SMR was statistically significantly less than in the Finnish population during the first 4 postoperative follow-up years in the metal-on-metal cohort and during the first 7 postoperative years in the non-metal-on-metal cohort.

**Table 4.** Observed and expected numbers of deaths, and standardized mortality ratios for the metal-on-metal and non-metal-on-metal cohorts in the main disease groups

	MoM cohort				Non-MoM cohort			
Cause of death	Obs	Exp	SMR	95% CI	Obs	Exp	SMR	95% CI
Cancer	114	173	0.66	0.55–0.78 **^c^**	764	873	0.87	0.81–0.93 **^c^**
Cardiovascular	131	196	0.67	0.56–0.78 **^c^**	1,274	1,719	0.74	0.70–0.78 **^c^**
Respiratory	11	25	0.44	0.22–0.78 **^b^**	84	204	0.41	0.33–0.50 **^c^**
Accidents and violence	45	46	0.97	0.71–1.29	180	158	0.82	0.69–0.97 **^a^**
All causes	365	562	0.65	0.58–0.71 **^c^**	2,785	3,846	0.72	0.70–0.75 **^c^**

MoM: metal-on-metal; Obs: observed number of deaths; Exp: expected number of deaths; SMR: standardized mortality ratio; CI: confidence interval.

**^a^** p < 0.05,

**^b^** p < 0.01,

**^c^** p < 0.001.

The SMR for cardiovascular deaths was 0.67 (CI: 0.56–0.78) in the metal-on-metal cohort and 0.74 (CI: 0.70–0.78) in the non-metal-on-metal cohort, relative to the Finnish population (Table 4). The SMR for cardiovascular deaths in a follow-up time of 5 years or more since operation was 0.81 (CI: 0.50–1.2) in the metal-on-metal cohort and 0.98 (CI: 0.90–1.1) in the non-metal-on-metal cohort relative to that in the Finnish population. The separately analyzed SMR for ischemic heart disease deaths in a follow-up time of 5 years or more since operation was 0.77 (CI: 0.40–1.34) in the metal-on-metal cohort and 0.90 (CI: 0.80–1.01) in the non-metal-on-metal cohort relative to that in the Finnish population. The risk of cardiovascular deaths in the metal-on-metal cohort was less than that in the non-metal-on-metal cohort (RR = 0.79, CI: 0.64–0.97; p = 0.02). Separately analyzed risk for ischemic heart disease deaths in the metal-on-metal cohort was not any higher than in the non-metal-on-metal cohort (RR = 0.78, CI: 0.60–1.02; p = 0.07).

**Figure:**
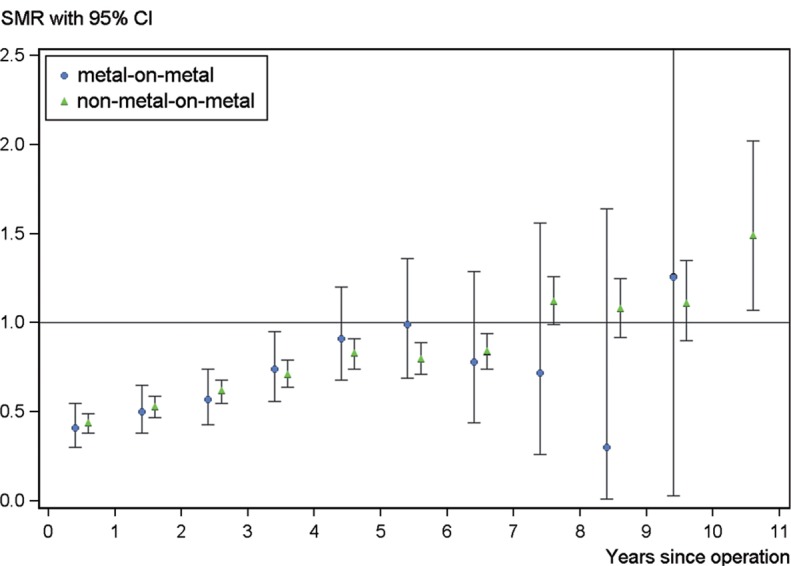
Standardized mortality ratios (SMRs) for deaths from all diseases in annual follow-up of metal-on-metal (MoM) and non-metal-on-metal (non-MoM) cohorts.

The risk of death from cancer in the metal-on-metal cohort was less than in the non-metal-on-metal cohort (RR = 0.78, CI: 0.63–0.97; p = 0.02). The risks of death from respiratory disease and of death from accidents and violence in the metal-on-metal cohort were similar to those in the non-metal-on-metal cohort (RR = 0.86, CI: 0.42–1.74; p = 0.7; and RR = 0.92, CI: 0.61–1.39; p = 0.7, respectively).

The only statistically significant interaction in the category regression analysis was that for implant type and sex in deaths from all causes (p = 0.01). The risk ratio for male patients in the metal-on-metal cohort was 0.70 (CI: 0.60–0.82) and for female patients in this cohort it was 0.98 (CI: 0.78–1.2).

## Discussion

The main finding of this study was that the overall risk of cancer was not higher than in the non-metal-on-metal cohort. This finding is in accordance with previous findings (Visuri et al. 1996, 2010a, Onega et al. 2006, Smith et al. 2012, Mäkelä et al. 2012, Brewster et al. 2013). Patient selection—i.e. the healthy-patient effect—had an appreciable influence on overall and site-specific risk of death during the first years of follow-up, both in the metal-on-metal and in the non-metal-on-metal cohorts. The overall and site-specific risk of death was not higher in the metal-on-metal cohort than in the non-metal-on-metal cohort, not even after the patient selection bias had ceased after the first 5 years of follow-up. The findings concerning risk of death agree with previously published results (Visuri et al. 1994, 2010b, Lie et al. 2000, Ramiah et al. 2007, McMinn et al. 2012). The risk of soft-tissue sarcoma and basalioma in the metal-on-metal cohort was higher than in the non-metal-on-metal cohort, as in our previous report (Mäkelä et al. 2012). 2 liposarcomas at the site of metal-on-metal hip replacement were found in 2011 in Finland. The risk of soft-tissue sarcoma and basalioma may be elevated by chance alone.

### Strengths and limitations of the study

One strength of the present study was the population-based design, with high numbers of patients with metal-on-metal hip implant. We were able to assess mortality according to cause of death, and to determine the incidence of different cancer types by combining data from the Finnish Arthroplasty Register and the Finnish Cancer Registry. One weakness was the short follow-up time. Another weakness was the lack of information on potential confounding factors regarding mortality and cancer risk. Furthermore, the supposed systemic complications from metal-on-metal replacements, such as cardiomyopathy, are rare. The ability of registry data to pinpoint deaths from cardiomyopathy may be inadequate.

### Comparison with other studies

The long-term overall risk of death was not increased using first-generation metal-on-metal hip implants (Visuri et al. 2010b). The 10-year life expectancy of conventional total hip arthroplasty patients is higher than that of the general population (Visuri et al. 1994, Lie et al. 2000, Ramiah et al. 2007). Male patients with metal-on-metal Birmingham hip resurfacing had a lower risk of death than those with a conventional hip device (McMinn et al. 2012). However, the Birmingham hip resurfacing patients were younger and healthier than those with a conventional hip replacement. These findings by McMinn et al. are in accordance with our data. Patient selection, i.e. the healthy-patient effect, probably explains a major part of the better survival of hip replacement patients compared to the standard population. Cardiovascular deaths due to cobaltism associated with metal-on-metal hip implants are rare and exceptional (Zyviel et al. 2013). Most arthroprosthetic cobaltism cases are probably curable when the implant has been revised, and cannot therefore be detected on the basis of mortality data.

The cancer risk of the patients with first-generation metal-on-metal total hip arthroplasty was not elevated, even in long-term follow-up (Visuri et al. 1996). Using hospital discharge, cancer, and mortality records, Brewster et al. (2013) studied the incidence of cancer in 1,317 metal-on-metal resurfacing arthroplasty patients in Scotland who were operated between 2000 and 2009. The risk of cancers overall (n = 39) was not increased (Brewster et al. 2013). Smith et al. (2012) studied 40,576 hip replacement patients with metal-on-metal bearing surfaces and 248,995 with alternative bearings, based on data from the National Joint Registry of England and Wales and hospital episode statistics. Compared to alternative bearings, there was no evidence that metal-on-metal bearing surfaces were associated with an increased overall risk of cancer (after a mean follow-up of 3 years). There was no increase in the risk of malignant melanoma or hematological, prostate, and renal tract cancers either. Furthermore, the overall cancer risk in our metal-on-metal cohort was not increased in our previous report covering patients operated during the years 2001–2010 who were followed until 2010 (Mäkelä et al. 2012). All these previous findings are in accordance with our current findings with a follow-up time until the end of 2011.

The risk of soft-tissue sarcoma in the metal-on-metal cohort was increased in our previous report, but not statistically significantly (Mäkelä et al. 2012). In the linkage study based on data from the National Joint Registry of England and Wales and hospital episode statistics (Smith et al. 2012), sarcoma risk associated with meta-on-metal hip replacements was not analyzed separately. In our work, 2 new sarcoma cases were diagnosed in the metal-on-metal cohort in 2011 after the previous analysis based on follow-up data until 2010. To our knowledge, the 2 liposarcomas diagnosed in 2011 in Finland are the first descriptions of liposarcoma at the site of a metal-on-metal hip implant. Stephensen et al. (1999) published a case report of a liposarcoma in the adductor lodge in a 57-year-old male rheumatoid patient with a conventional total hip arthroplasty. However, the total number of sarcoma cases in our study was small. The increased incidence of sarcomas in the metal-on-metal cohort may still be a chance finding. Hundreds of thousands of metal-on-metal hip replacements have been performed worldwide, but only 5 malignant local tumors at the site of first-generation metal-on-metal replacements have been reported previously (Visuri et al. 2006).

A risk of melanoma has been associated with conventional total hip arthroplasty in some of the earlier studies (Nyren et al. 1995, Olsen et al. 1999, Visuri et al. 2003, 2006) but not all of them (Visuri et al. 2010a). Incidence of melanoma was found to be increased in patients with a conventional hip implant inserted during 2005–2009 in Scotland (SIR = 1.4, CI: 1.1–1.9), but not in patients with a metal-on-metal hip resurfacing device (Brewster et al. 2013). The incidence of melanoma in the non-metal-on-metal cohort in the current study was higher than in the Finnish general population, as also found in our previous report (Mäkelä et al. 2012). Melanoma risk in the metal-on-metal cohort was similar to that in the non-metal-on-metal cohort. The increased incidence of melanoma in the non-metal-on-metal cohort may have been due to survey bias. In the study based on the National Joint Registry of England and Wales, the risk of melanoma was not higher in metal-on-metal patients than in patients with other bearing options in the first 7 years after arthroplasty (Smith et al. 2012). However, the assessment of outcome was based on linkage to hospital episode statistics, which may have been associated with less quality assurance than data from a cancer registry. Information on some cancers may have been missing, e.g. cutaneous melanoma, which does not necessarily lead to hospital admission (Smith et al. 2012, Brewster et al. 2013).

The metal-on-metal cohort showed a higher risk of basalioma than the non-metal-on-metal cohort, which is in accordance with our previous report (Mäkelä et al. 2012). Basalioma incidence was increased in the patients with a conventional hip implant inserted during 2005–2009 in Scotland (SIR = 1.1, CI: 1.0–1.2), but not in patients with a metal-on-metal hip resurfacing (Brewster et al. 2013). In other previous studies on total hip replacement patients, basalioma was either not registered at all or was included in the category of other skin cancers (Visuri et al. 2006, Smith et al. 2012). In theory, levels of metal ions in the skin could be elevated after metal-on-metal hip replacement, perhaps causing DNA damage together with ultraviolet radiation. However, we are not aware of any studies that have been conducted to address this issue.
